# Prevention—The Technique of the Future

**Published:** 1915-09

**Authors:** C. H. Gerrish

**Affiliations:** Exeter, N. H.


### PREVENTION—THE TECHNIQUE OF THE FUTURE.

DR. C. H. GERRISH, EXETER, N. H.

Some fifty years ago a patient of Dr. Riggs' came to me for treatment of pyorrhea, asking me to treat her mouth and teeth on the same lines as pursued by Dr. Riggs. It was a revelation to me what a *clean* root, a polished root, meant! This inspired me to extend the cleansing process to the crowns of the teeth, reasoning that if pyorrhea was checked or cured thereby, why might not also caries of the teeth be stopped or prevented in that manner? Hence, upon the theory that a polished tooth might be immune from decay, I began what has proved to be my life-work—meeting with much opposition and the superstition that touching the enamel of the teeth meant decay and destruction. No precedent had I to go by, as the polishing of teeth was then an unknown field of dental art.

Having started with the use of the orangewood stick and pumice-stone, Arkansas pencil, linen tape, quill picks, thin linen tape, and waxed floss silk, all by hand, I am using at the present time the Arkansas stones, rubber disks, brush-wheels, etc., in the dental engine; and the results have been marvelous. My theory was sound and correct, as the vital facts now show; in some of my oldest patients I have not been obliged to make a single filling in an average of twenty years; others have been immune for over twenty-five years.

### Clinical Results of Immunity to Caries.

My position regarding the matter is simply this: Either this technique is valuable, or a delusion, or I am an impostor. There is no middle ground for me to fall back upon—and I desire none. My credentials are my patients, and open to your inspection. To quote from a letter of Dr. Rogers and Dr. Libby of Boston, who examined the teeth of one of my patients: "We should judge by the splendid condition of her teeth, especially at the gingival line, and the general condition of her gums, that she is safe for another twenty-five years." This patient has not a single filling anterior to the molars upper or lower. Were this an isolated case, one could ascribe it to heredity, but what about another patient who has had every tooth filled, yet has been immune from recurrent caries for twenty years? These results have been obtained in patients who have been in my care from childhood, and who have followed my directions to the letter. Can these results be obtained in all cases? No; degenerates and those deficient in their heritage are exceptions, but in every one of even these cases the treatment will produce a delay in the decay, and prolong the life and use of the teeth.

### The Writer's Technique.

What is the technique? It begins with the mother, then with the child's temporary teeth, and as soon as the permanent teeth erupt and polishing is continued with stones none of which are of coarser grit than the Arkansas stones, and refined silex. The time required for treatment ranges from an hour or two in children to four or five hours in the grown-up. As for frequency, the treatment is given from one to four times a year.

As the result of this polishing, we secure a smooth enamel surface; the lines of imbrication are worn down in time, from five to ten years being required to secure this result. Too deep cutting or polishing is fatal, as is the use of coarse stones. The final finish is obtained with a mixture of prepared chalk and oxid of tin in the proportion of three to one, which leaves the enamel surface as smooth as a diamond.

### The Patient's Part.

The patient's part consists, in the morning, in brushing the teeth, giving them a so-called "dry brush"—that is, the brush is just moistened, covered with powder, and used with a rotating movement, until the gelatinous plaques are removed. *Paste will not do this,* or, to be less emphatic, not for *my* patients, because they have tried it. At night a dental cream can be used to great advantage, the last step consisting in the use of waxed floss.

### Effect of Treatment.

What change, if any, takes place in the enamel structure under this intensive polishing, continued for fifty years, I do not know; I can only voice my conviction—and, not being a scientific man, my views may be guesswork. Apparently, the effect is stimulating, and I think the enamel undergoes a change, though the authorities are against me. However that may be, I get the same results that seem evident in erosion, namely, immunity from decay without the waste of tooth material.

### Gutta-percha Fillings.

The filling that saves a tooth from further decay must be a perfect stopping. All stoppings are fillings, but all fillings are not stoppings. Gutta-percha is the ideal stopping, especially red base-plate gutta-percha, because it contains a germicide and it adapts itself to the cavity; besides, it is forever swelling. It swells, and it also smells, and when properly used is quite permanent in its results.

I recently refilled a tooth which had been kept perfect for twenty-three years by a gutta-percha stopping except for the wearing away of the filling. Non-cohesive gold, among the many forms of gold, possesses the same characteristics, and I am today watching fillings which were inserted over fifty years ago, and which preserve the teeth and look as well, save for a little wear, as when they were introduced.

### Accessories.

Among the aids that I have found to be of benefit are the Indexo brush, which is a rubber cap with raised papilla used on the forefinger to produce general massage of the teeth and gums. Should there be an inflamed condition, a saline solution, as suggested by Dr. Libby, is very efficacious in bringing about a normal state. I also recommend the daily use of Johnson's "Educators," a hard-baked whole-wheat cracker which not only furnishes the materials of tooth structure, but tends to the formation of a perfect arch, as it requires much force to masticate. In cases of extreme sensitiveness of the tooth structure at the gingival margins, I have found Phillips' milk of magnesia a specific; it has also proved very efficacious in the prevention of decay.

### Incipient Caries and Pyorrhea Prevention.

Incipient caries, especially at the gingival margins, I treat on similar lines, viz., with the use of Arkansas stones by the method above described, leaving the formerly carious portion thoroughly polished, and establishing immunity for one year. If this method is followed up, with the co-operation of the patient, this immunity can be prolonged to ten and twenty years. Other results follow this mechanical smoothness of the tooth structure. Pyorrhea is unknown; I have never had a case among patients who have been subjected to this ideal treatment.

### A Plea for Prevention.

As a profession we have accomplished wonderful results in the treatment of diseases and in artistic fillings, and crown and bridge work. Yes!—fillings that do not stop decay, crowns that do not fit, bridges that smell to heaven! With the coming of the Forsyth Infirmary and the Evans Institute, two of the grandest blessings to mankind, let the lessons of prevention take rank with, or rather precede, all other teachings. May the profession, which is doing such grand preparatory work for the student and sending him out to battle with disease and decay, take this word from one who has devoted his life to prevention, and who is obliged, but who is also glad, to stand at the chair after fifty years of service. May they teach the students that the best filling is *no* filling. To save *from* is far better than to save *by*. For the time is coming, and coming rapidly, when prevention, the grandest word in the English language, will and must prevail in dentistry, medicine, law, religion, and national life. The temple is nearly completed, but one stone is lacking to restore the arch, the keystone, the stone rejected of you builders, on the face of which will be written "Prevention."

In conclusion let me quote from Dr. Bloodgood, of Baltimore—who, in his closing remarks before the National Dental Association, said: "The great majority of dentists prefer to do the more expert mechanical work, bridge work and other things that require great skill. *They don't like to clean the teeth.* (Italics mine.) The day is coming when more people's teeth will be saved by keeping the people's teeth clean than by doing bridge work! Again, how many cases of Bright's disease, that shortens the lives of many great men and women, have their portal of entrance through the teeth? So this thing you dislike to do, cleaning the teeth, may be the most important and expert thing you can do. I believe it is an expert thing."

If I can induce any of the younger practitioners to take up this work of prevention, it will gladden my heart. I trust you will pardon me if, in conclusion, I read you my creed: "If cleanliness is next to godliness, or goodness, then begin and end the day with a prayer, followed by a thorough cleansing of the mouth and teeth, thus rendering yourself immune from deceit and decay, the devil and disease;—for a clean soul and a clean mouth are much to be desired."—*Cosmos.*

